# Bivariate segmentation of SNP-array data for allele-specific copy number analysis in tumour samples

**DOI:** 10.1186/1471-2105-14-84

**Published:** 2013-03-05

**Authors:** David Mosén-Ansorena, Ana María Aransay

**Affiliations:** 1 Genome Analysis Platform, CIC bioGUNE & CIBERehd, Technologic Park of Bizkaia, Building 502, 48160 Derio, Spain

## Abstract

**Background:**

SNP arrays output two signals that reflect the total genomic copy number (LRR) and the allelic ratio (BAF), which in combination allow the characterisation of allele-specific copy numbers (ASCNs). While methods based on hidden Markov models (HMMs) have been extended from array comparative genomic hybridisation (aCGH) to jointly handle the two signals, only one method based on change-point detection, ASCAT, performs bivariate segmentation.

**Results:**

In the present work, we introduce a generic framework for bivariate segmentation of SNP array data for ASCN analysis. For the matter, we discuss the characteristics of the typically applied BAF transformation and how they affect segmentation, introduce concepts of multivariate time series analysis that are of concern in this field and discuss the appropriate formulation of the problem. The framework is implemented in a method named CnaStruct, the bivariate form of the structural change model (SCM), which has been successfully applied to transcriptome mapping and aCGH.

**Conclusions:**

On a comprehensive synthetic dataset, we show that CnaStruct outperforms the segmentation of existing ASCN analysis methods. Furthermore, CnaStruct can be integrated into the workflows of several ASCN analysis tools in order to improve their performance, specially on tumour samples highly contaminated by normal cells.

## Background

Two chief genetic instabilities associated to tumoural cells are genomic copy number alterations (CNAs) and somatic loss of heterozygosity (LOH) events, which represent a deviation from the normal allele-specific copy numbers (ASCN). Both imbalances have been reported to affect the expression of oncogenes and tumour-suppressor genes [1], and therefore, the accurate characterisation of ASCNs in tumoural samples is critical in order to identify candidate cancer-related genes, to discriminate cancer types [2] and to understand tumour initiation and complexity [3].

Single nucleotide polymorphism (SNP) arrays of Illumina [4] and Affymetrix [5] platforms allow screening for ASCNs at high resolution and throughout the whole genome by providing measures for the log R ratio (LRR), which reflects the total intensity signals for both alleles, and the B allele frequency (BAF), which is the relative proportion of one of the alleles with respect to the total intensity signal. Both LRR and BAF signals are required for a complete characterisation of ASCNs since they provide complementary information. Yet, although each combination of copy number and allelic ratio has an expected LRR value and a specific BAF pattern, these signals can be blurred due to experimental probe-specific noise and by autocorrelated [6] and dye [7] biases, respectively.

In the study of ASCNs over tumour samples with SNP arrays, three additional issues need to be considered. First, there is a LRR baseline shift that depends on the ploidy of the sample. Second, tumour biopsies can be contaminated with normal cells, whose genotypes are mainly diploid, which make the LRR and BAF signals to shrink and converge towards those of a diploid state proportionally to the degree of contamination [8]. Third, tumours can be composed of several subclones, this is, subpopulations of cells that harbour specific alterations along with the shared ones, which makes LRR and BAF signals even more complex [9]. The second and third tumour-specific issues, together with the experimental noise and biases, affect the ability to correctly delimit regions with different ASCNs. Therefore, inferring change-point locations from tumour samples requires mathematical models whose performance is affected as little as possible by these issues.

Two approaches are used for the detection of ASCNs in tumour samples on SNP arrays, both of which inherit from methodologies applied to aCGH. The most recurrent approach is based on a combination of a hidden Markov model (HMM) and an expectation-maximisation (EM) algorithm. OncoSNP [10] and GPHMM [11] are two recent HMM-based tools validated on Illumina data which, in contrast to previous methods [12-14], are capable of estimating both normal cell contamination and LRR baseline shift. Most existing HMM-based methods, including the two aforementioned ones, integrate the LRR and BAF signals into the same model, which confers them more change-point detection power. Yet, the pre-established levels of HMMs are not prepared to characterise the observed continuous mean levels that arise due to the presence of multiple subclones [9,15]. Additionally, HMMs require parameterisation on region probability and length, which vary among samples and are not known a priori. Arguably due to the aforementioned issues, in a recent method comparison [16] HMM-based methods were outperformed by a change-point detection method. For this reason, we propose tackling the problem of ASCN analysis from a change-point-based stand.

Methods based on change-point detection algorithms are typically comprised by segmentation followed by a calling step [17,18]. This approach does not assume pre-established signal levels and does not require parameterisation of a priori knowledge. Two change-point-based approaches for unpaired tumour samples that use both LRR and BAF signals have been developed: GAP and ASCAT. PSCBS [19] also falls into this category, but it only works on coupled tumour samples and does not automatically estimate normal cell contamination. In the segmentation step, GAP segments the LRR and BAF signals independently and merges the change-points with those that come from the detection of LOH germline regions in BAF. On the contrary, ASCAT performs a single bivariate segmentation instead of two univariate segmentations, because the integration of the signals into the same formulation can increase the power to detect dimmer joint changes and reduce false positives. However, the extension from the univariate to the bivariate case is not trivial and depends on the characteristics of the considered segmentation approach, which may fall into one of two broad categories: boundary-based and region-based (see [20] for an analogue distinction in image segmentation).

In the boundary-based differential approach, change-points are seen as inflection points, this is, places where the first derivative has local extrema. Only local information around each point is used to compute the derivative, often resulting in spurious and merged change-points. Multiresolution analysis can be performed by computing the derivative at various window sizes, but region-based approaches are the most adequate to obtain more information for segmentation decisions, although they sacrifice change-point location accuracy. Region-based approaches can be broken down into segment-growing, split-and-merge and global optimisation. Region-growing starts with a number of random single-point regions. Neighbouring points are added to a region if they are similar enough, according to a certain homogeneity criterion; otherwise, a new segment is started. A representative example of split-and-merge is the binary segmentation, which selects as a change-point the position that divides the data into two segments with the most different means. The process is recursively applied to each segment until it cannot be divided into two subsegments with a mean difference that is significant enough. Then, similar regions are merged back together following some pruning criterion. Circular binary segmentation (CBS) [21] is a modification that allows at each step for the detection of one change-point or two, where the subsegment in the middle has a different mean than the other two subsegments. Global optimisation methods try to optimise an objective function, called cost function in minimisation and utility function in maximisation. Some methods, such as the structural change model (SCM) [15,22], return the actual optimum. Others, namely heuristics, perform a non-exhaustive scan over the combinatorial space of change-points and can thus be trapped into local extrema.

Current change-point detection methods [17-19] are based on region-based segmentation algorithms, which are more adequate for ASCN analysis because finding change-points is more important than establishing their accurate location. More precisely, GAP is based on CBS and ASCAT on bivariate global optimisation. PSCBS, aimed at paired tumour samples, and BAFsegmentation [8] and TAPS [23], which only segment either BAF or LRR, are also based on CBS.

The application of the univariate segmentation methods to the bivariate data from SNP array requires: (i) knowing how the transformation typically applied to the BAF signal influences the applicability of certain segmentation methods and their extension to the bivariate case, and (ii) a mathematical model that generalises the extension from the univariate to the bivariate case. We provide such formalisations, illustrate that the approach taken by ASCAT is a specific case of the bivariate generalisation and discuss why there are more suitable formulations of the bivariate segmentation for ASCN analysis. Then, we show how the bivariate framework is applied to the SCM model in order to achieve CnaStruct, a method that outperforms the segmentation of existing approaches.

## Methods

### BAF transformation and characterisation

**Figure 1:**
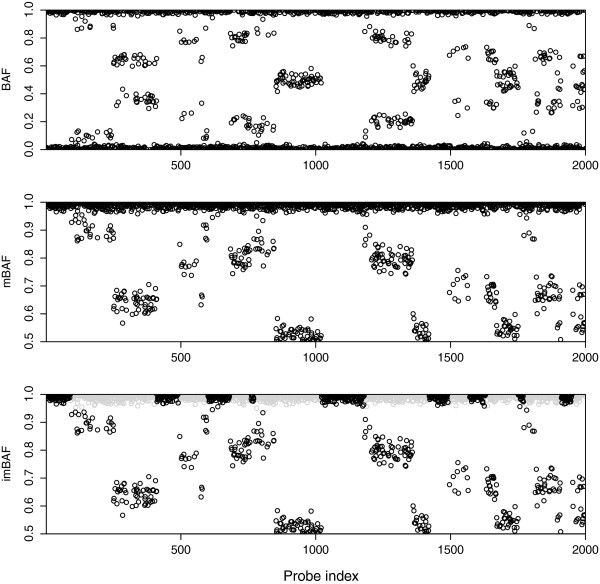
**Transformation from BAF to mBAF and then, into imBAF.** A sample toy BAF signal is transformed to mBAF and then into imBAF. X-axis: probe index. Y-axis: B allele frequency (first) and B allele frequency mirrored along the 0.5 axis (second and third). Grey points represent homozygous SNPs within heterozygous regions.

Methods for the detection of changes in mean on univariate data can be extended to the bivariate case in order to be applied jointly to LRR and BAF, called “variables” from here on. However, a transformation of the BAF variable, which leaves a mostly single-banded signal along the genomic axis, is preferred for posterior segmentation. For the matter, BAF is first mirrored along the 0.5 axis in order to obtain mirrored BAF (mBAF). Then, non-informative SNPs, defined as those in homozygous bands of heterozygous regions, are removed, leaving a transformation that has already been described [8,17] but not named, so we call it informative mirrored BAF (imBAF) (Figure 1).

The resulting imBAF is not homoscedastic for two reasons: (i) the homozygous band resembles a mixture of a point mass function and a truncated normal distribution with lower variance than its heterozygous counterparts; (ii) the distribution of the heterozygous band, when near the 0.5 axis, is truncated due to the mirroring and thus has lower variance. Nevertheless, homoscedasticity violations seem to be sufficiently small so as to not impact segmentation performance of the approaches we assessed (CBS and SCM).

Non-polymorphic probes yield missing values on the BAF variable. Additionally, the transformation of BAF into imBAF generates more missing values, all of which can be easily removed for the application of univariate segmentation approaches. However, the removal of missing values on bivariate approaches typically implies the exclusion of the corresponding LRR observations and, thus, loss of information. Therefore, missing values should be either handled by the segmentation method or imputed, which can be easily done through interpolation. In general, we observed that constant interpolation is more adequate for change-point detection than linear interpolation, because this latter inserts values that lie between imBAF bands, distorting the profile.

### Bivariate segmentation

The methodology of univariate change in mean segmentation can be generically formalised in the following way. Consider the energy value *δ* of a generic event that arises in a segmentation process, subject to fitness assessment through a decision function *τ*, given a parameterisation *θ* (Equation 1). In region-growing, *δ* can be the difference between a segment’s measure of centrality and the value of a neighbour observation. The Boolean function *τ* decides whether *δ* is small enough to incorporate the new point to the growing segment given, for instance, a threshold that depends on the length and variance of the segment. In binary segmentation, *δ* can be the student’s t (or its corresponding p-value) that arises from testing the difference between the left and right subsegments, whereas *τ* is a simple thresholding function. Other typical *δ*’s are residual sums of squares (RSSs) and values of peaks in a derivative signal.

(1) $$\begin{array}{c} \tau :R\to B\\ \tau \left(\delta |\theta \right) \end{array}$$

**Figure 2:**
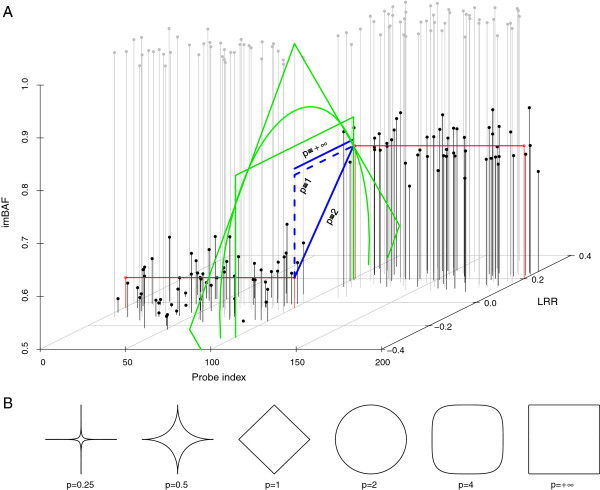
**Minkowski distances. **(**A**) Example with Minkowksi distances for the case where *δ* represents a difference of segment means. Each point represents the value of a probe for the LRR (y-axis) and imBAF (z-axis) variables at its corresponding index (x-axis). Black dots: informative SNPs. Grey dots: non-informative SNPs. Red lines: segment means calculated from informative SNPs. Blue lines: Minkowski distances, between the two segments, of order 1 (the sum of the dashed lines, which correspond to *δ*_*LRR*_ and *δ*_*imBAF*_), 2 and infinite. Green shapes: lines that delineate mean differences with the same Minkowski distances of orders 1 (rotated rectangle), 2 (oval), and infinite (rectangle), with respect to the first segment mean. (**B**) Shapes in the bidimensional space of Minkowski distances of different orders.The points that make up each shape are, from the shape’s centre, at an equal *p* -order Minkowski distance.

The generalisation can be extended to the multivariate case, where the objective of segmentation ramifies into finding recurrent changes in mean or changes present on a subset of variables. Approaches that detect points where the variables change together are based on the change in the covariance structure. However, we also seek to detect points where the variables LRR and imBAF change in the opposite direction and where just one of them suffers a relevant mean change. The reason is that the copy number may remain constant along two segments with different allelic ratio, and vice versa. This takes us to the adequate model for our problem: a bivariate change in mean. Here, the bivariate decision function *τ* arises from jointly applying the function over the *δ*'s of the two corresponding variables, balanced with a normalisation constant *β*. This is essentially an averaging process, where the variable that is expected to provide more information is given more weight. Exponentiation before the averaging promotes the importance of changes present in just one of the variables. The resulting overall *δ* is a metric in the Euclidean space $R^{2}$ called Minkowski distance, whose parameter *p*, a real positive number, determines its order and models the interaction between the variables *V*1 and *V*2, which correspond to LRR and imBAF in our case (Equation 2, Figure 2A). Letting *p* = 1 (e.g. the approach taken by ASCAT) means that events in just one of the variables, represented by the corresponding variable *δ*, need to be more extreme than joint events in order to be considered relevant by *τ*. By contrast, Minkowski distances of order *p* > 1 promote, through the exponentiation, the importance of *δ* values in which there is greater disequilibrium between their variable *δ*'s, so changes in a single variable are easier to detect. The extreme case *p* = ∞ is equivalent to making the decision based on the maximum weighted variable *δ* (Equation 3). An adequate order *p* is defined then as the one that keeps a balance in the filtering of joint and single events. In general, orders greater than 1 are appropriate for maximisation problems and lower than 1 for minimisation problems.

(2) δ=δV1p+β*δV2p1p

(3) τδV1∞+β*δV2∞1∞|θ=τmaxδV1,β*δV2|θ

### CnaStruct

#### The model

The SCM segmentation [15,22] is a region-based, global optimisation approach that models the data as a piecewise constant function:

(4) $$z_{k}=\mu_{s}+\varepsilon_{k}\;\text{for}\;t_{s}\leq k<t_{s+1}$$

where *k* = 1…*n* indexes the observations of the variable *z*, *t*_1_…*t*_*s*+1_ parameterise the borders of the *S* segments, *μ*_*s*_ is the mean value of the *s* -th segment and ? _*k*_ are the residuals.

CnaStruct is based upon SCM and extends it to a bivariate form that is suitable for ASCN analysis on SNP-array data. For the description of the bivariate form, consider first the residual sums of squares (RSSs) of a segment *s*, with borders *t*_*s*_ and *t*_*s*+1_, in the LRR and imBAF variables respectively:

$$\text{rs}s_{r,s}=\overset{t_{s+1}-1}{\underset{k=t_{s}}{\sum }}{\left(r_{k}-{\hat{\mu }}_{s}\right)}^{2}\;,\;\text{rs}s_{b,s}=\overset{t_{s+1}-1}{\underset{k=t_{s}}{\sum }}{\left(b_{k}-{\hat{v}}_{s}\right)}^{2}$$

where *r*_*k*_ and *b*_*k*_ are the LRR and imBAF observations at the indexed SNP probe *k*, and

$${\hat{\mu }}_{s}$$

and

$${\hat{v}}_{s}$$

are the mean values of the segment in the two variables. Following the notation introduced for bivariate segmentation, each *δ* is linked to a segment *s* through the Minkowski distance given its RSSs in the two variables:

δs=rssr,sp+β*rssb,sp1p

SNP-array data may contain missing values in the LRR and BAF variables and, in addition, the transformation from BAF results in a high percentage of missing values in imBAF. Such cases do not contribute to the corresponding RSS and thus *δ*_*s*_ should be normalised with respect to the number of actually observed values in each variable:

δs=lr,slr,s+lb,s*rssr,sp+lb,slr,s+lb,s*β*rssb,sp1p

where *l*_*r*,*s*_ and *l*_*b*,*s*_ are the number of non-missing observations of a segment *s* present in the LRR and imBAF variables, respectively.

Under the bivariate SCM, the model in Equation 4 is fitted by minimising the following cost function, which is the sum of all segment *δ*'s:

$$G\left(t_{1}\ldots t_{S}\right)=\overset{S}{\underset{s=1}{\sum }}\delta_{s}$$

A dynamic programming algorithm (see [24]) finds the optimal set of change-points *t*_1_…*t*_*S*_. The decision function *τ* is defined recursively in such dynamic programming algorithm, where it allows a *δ*_*s*_ if the segment *s* minimises the segmentation cost with *x* segments, given the cost of the best segmentation with *x* − 1 segments up to the beginning of *s*:

$$\tau \left(\delta_{s}|\theta \right)=\left(\text{min}G\left(t_{1}\ldots t_{x-1}\right)+\delta_{s}=\text{min}G\left(t_{1}\ldots t_{x}\right)\;|\theta \right)$$

where *θ* establishes constraints for the number of segments and the maximum allowed segment length. This last constraint reduces computational time complexity from O(n^2^) to O(nl) [15].

Because this is a fitting problem, Minkowski distances of order *p* < 1, which model the interaction between the variables as a decay function, are appropriate (Figure 2B). Such approach makes a pair of strong-weak fits result in a lower cost than two average fits, given the same linear combination of residual errors. In other words, it promotes the detection of strong mean shifts albeit in a single variable, such as transitions between segments with same allelic ratio but different copy number. The weighting coefficient *β* parametrises the relative contribution of the imBAF variable to the cost function. For a balanced contribution, its value should quantify the ratio of informative observations in each variable and the relationship between their signal-to-noise ratios. While the standard deviation is around 6 times larger on LRR independently of the sample data, the expected mean changes depend on the amount of LOH regions and the diversity of copy numbers among other sample factors. The tests we have performed on synthetic data suggest establishing a weighting between variables of *β* = 1, and show that a Minkowski distance of order of *p* = 1/4 captures very well the interaction between the variables.

#### Model selection

Data can always be fitted better by increasing the number of change-points *S*, so there is a need to find the optimal *S*. An option is the use of penalised log-likelihoods [22].

Assuming that the residual errors ε _*k*_ in Equation 4 are independent, the log-likelihood of a model using the Bayesian information criterion (BIC) is:

$$\log{\tilde{L}}_{\text{BIC}}=-\frac{N}{2}\left(1+\text{log}\frac{2\pi \delta }{N}\right)-k*S*\text{logN}$$

where *N* is the number of observations, *S* is the number of segments, *δ* determines the best segmentation with *S* segments and *k* = 1. Huber et al. [15] discuss that log-likelihood penalisation overestimates the number of change-points in transcriptional data. In SNP-array copy number data, we found the BIC-penalised log-likelihood to be a satisfactory model selector, but it can be adjusted depending on the desired sensitivity required for downstream analyses.

#### Software

We built a CnaStruct R package that is freely available at http://web.bioinformatics.cicbiogune.es/cnastruct. The segmentation function is based on the one included in another R package, called tilingArray [15]. Maximum segment length and the number of maximum segments are parameters inherited from such function. The order of the Minkowski distance and the weighing constant between variables are also parameterised, with *p* = 1/4 and *β* = 1 set as the default values. Finally, CnaStruct allows the selection of *k* in the information criterion (default is *k* = 1, BIC), in order to alter the number of located change-points.

## Results and discussion

We evaluated the performance of CnaStruct against the two latest HMM-based methods (GPHMM [11] and OncoSNP [10]) and the two change-point detection methods (ASCAT [18] and GAP [17]) that use both LRR and BAF variables.

All the assessed methods can handle Illumina data, so we evaluated them on the benchmarking dataset from Mosén-Ansorena et al. [16], which simulates data from this platform. The dataset considers five characteristic tumour alteration patterns (near-diploid, near-triploid, near-tetraploid, LOH-enriched and complex) and contains one hundred samples per pattern. Fragments with copy numbers 1, 2, 3, 4 and 5 with and without somatic or germline LOH spanning 10, 20, 40, 80 or 160 SNP probes were included and samples were generated at four percentages (0, 25, 50 or 75%) of non-tumoural cell contamination. Longer regions were not included because no major performance differences were expected from the longest considered region on (see Discussion for rationale on this matter). For a more detailed description of the datasets, see Mosén-Ansorena et al. [16].

A true change-point was considered recalled if at least one predicted change-point falls within a window of 3 probes from it, a threshold that is wide enough to recover most of the correct predictions in the benchmark dataset. Furthermore, from such window on, between-method differences do not vary significantly. Given that GAP outputs the result of merging three segmentations, the calculation of the specificity does not penalise repeated calls of the same change-point in order not to deflate its specificity.

**Figure 3:**
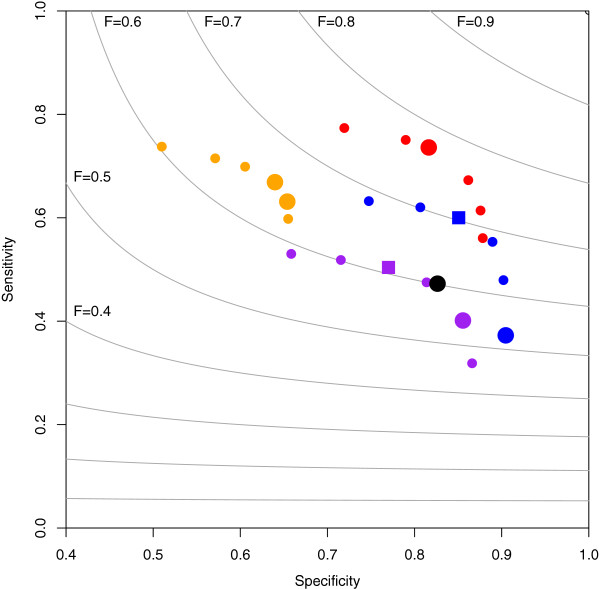
**ROC curves for the different methods over a subset of the synthetic data.** ROC curves that arise from running methods with different sensitivity parameterisations over the complex-patterned samples with 50% normal cell contamination. The combinations of pattern and contamination level were chosen for being representative of the overall performance. Sensitivity is shown in the vertical axis and specificity in the horizontal axis. Colour code: purple (ASCAT), red (CnaStruct), orange (GAP), black (GPHMM), blue (OncoSNP). Bigger dots correspond to the results obtained with default parameterisations (two for GAP due to the different parameterisations of CBS in the two versions of GAP). If applicable, squares correspond to the best non-default parameterisations. Grey lines are F-measure isocurves (the F-measure integrates, with the same weight, sensitivity and specificity in a single value).

Receiver operating characteristic (ROC) curves allow visual assessment of method performance and the influence of sensitivity parameterisation (Figure 3). To keep the consistency with the terms used in this text, we built ROC curves with specificities instead of false positive rates (FPR, 1-specificity). GPHMM does not provide any means of adjusting change-point detection sensitivity, so we could not evaluate its behaviour in these terms. The rest of the methods present dissimilar ROC curve shapes which reach varying specificity and sensitivity limits. The greatest difference is seen on sensitivity, where CnaStruct and GAP are clearly the methods that reach higher levels. However, given comparable change-point sensitivities, GAP has significantly lower specificity than the rest of methods. A reasonable explanation is that a bivariate model, which GAP lacks, prevents many false positives thanks to the additional information available at each point. Overall, CnaStruct combines the high sensitivity of GAP and the high specificity of the methods with a bivariate model.

The default parameterisations in OncoSNP and ASCAT are aimed to the detection of longer regions than the ones included in the analysed synthetic samples, so, in order to account for parameterisation differences and keep further comparisons fair, we replaced the default sensitivity-related values with those that achieved the best combination of specificity and sensitivity in the corresponding ROC curves. Such combination is called F-measure, the harmonic mean of specificity and sensitivity. However, notice that the traditional F-measure gives the same importance to both measures, which may not be adequate, as it has been noted that sensitivity is preferable over specificity [17]. Certainly, regions with the same allele-specific copy number can be merged a posteriori after excessive segmentation, but missed region borders cannot be recovered if too few change-points have been detected. Hence, long regions are easily identified regardless of parameterisation, but delimiting short regions requires high sensitivity. Because of this, (i) if a posteriori region merging is applied, parameterisations (of the same method) that prioritise sensitivity achieve better results on shorter regions while having similar results on longer ones (see Additional file 1 for an example), and (ii) CnaStruct’s downstream improvement is expected to be greater with respect to ASCAT, which mainly delivers lower sensitivity, than with respect to GAP, which delivers lower specificity.

**Figure 4:**
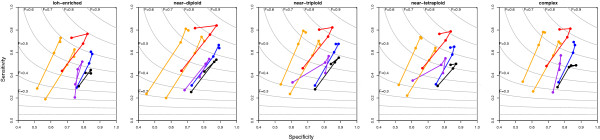
**Change-point sensitivity (y-axis) and specificity (x-axis) by sample pattern.** Change-point sensitivity (y-axis) and specificity (x-axis) by sample pattern. Dots connected by a line correspond to the sensitivity and specificity achieved by the corresponding method at the following normal cell contamination levels: 0%, 25%, 50% or 75%. Colour code: purple (ASCAT), red (CnaStruct), orange (GAP with CBS parameterisations from: original (left); updated (right)), black (GPHMM), blue (OncoSNP). Grey lines are F-measure isocurves (the F-measure integrates, with the same weight, sensitivity and specificity in a single value).

We ran the five methods with their optimal parameterisations based on their ROC curves and F-measures, with the exception of GPHMM, which does not allow parameterisation tuning. GAP was run with its default segmentation parameterisation in its original and updated version, which achieved similar F-measures. CnaStruct consistently achieves the best change-point sensitivities and F-measures out of the compared methods along the five alteration patterns and four normal cell contamination levels (Figure 4). Notice how the improvement in sensitivity is more noticeable at 75% contamination (lower points of the “sticks”) with respect to GAP, and at the rest of contamination levels with respect to ASCAT.

**Figure 5:**
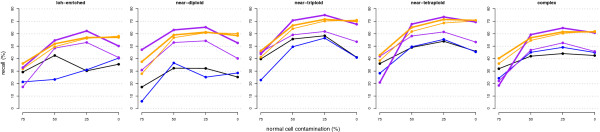
**Recall rates by normal cell contamination and alteration pattern.** Recall rates (y-axis) of each of the assessed methods, calculated by normal cell contamination (x-axis), over each of the five sample patterns. Colour code: purple (ASCAT), orange (GAP), black (GPHMM), blue (OncoSNP). Thicker lines correspond to the workflows in which CnaStruct was integrated.

To test whether downstream characterisation of allele-specific copy numbers improves with CnaStruct segmentation, we replaced the segmentation algorithms in GAP and ASCAT with CnaStruct (see Additional file 2). Then, we compared the new workflows against the original ones and the HMM-based approaches of GPHMM and OncoSNP. The results show that overall performance is improved in both cases (Figure 5). In the case of GAP, there is a slight drop at the null contamination level, but a relevant improvement under heavy contamination. In ASCAT, the improvement is even more significant, as expected, with a gain of around 20% in the recall rates of alterations that span 10 and 20 probes up to 50% normal cell contamination (Additional file 3). However, at 75% contamination, both the original and the CnaStruct-ASCAT workflows deliver highly variable results due to problems in the pattern recognition process of ASCAT. As a sidenote, we were surprised by how OncoSNP’s good performance on change-point detection was not translated to better alteration recall rates. There is not an overall best performer; instead, we see that the combined workflow of CnaStruct and ASCAT is best for samples with some contamination and the combined workflow of CnaStruct and GAP is best for samples without contamination (e.g. cell-line samples) or heavily contaminated. Given that contamination is significant but a priori unknown in many samples, CnaStruct-GAP is the most prudent choice.

Although we only assessed CnaStruct on Illumina-like data, we ran it in combination with GAP, ASCAT and TAPS on samples from either the Illumina or Affymetrix platform (Additional file 4). Visual assessment (see Additional file 5) shows a good performance, although ASCAT fails at the calling step on the sample with 53% of normal cell contamination. Furthermore, we hypothesise that the improvement with respect to existing methods for the Affymetrix platform is even greater than on the Illumina counterpart, given that the noisier profile of Affymetrix SNP-array data is more appealing for the bivariate segmentation and current methods for Affymetrix data, including GAP, only perform univariate segmentation. Particularly, TAPS [23] is an ASCN analysis tool for the Affymetrix platform whose change-point detection step consists on a simple CBS segmentation over the LRR variable, whose baseline shift is not automatically estimated. Still, when compared to GAP, it delivered a better performance [23]. Given that, as the authors state, the CBS segmentation in TAPS can be replaced by other approaches, we propose the combined use of CnaStruct and TAPS (see Additional file 2) for ASCN analysis on the Affymetrix platform. Given a proper construction of LRR and BAF, we believe that CnaStruct is a sensible segmentation method for high-throughput sequencing (HTS) ASCN analysis too, where, at the moment of writing, the only method that uses BAF [25] does not perform bivariate segmentation.

## Conclusions

We have first identified the issues that arise on segmentation due to imBAF characteristics, namely high value missingness and heteroscedasticity. Although such transformation had already been described, no literature existed on how imBAF’s peculiarities affect segmentation, and more specifically bivariate segmentation.

Then, we have introduced and formalised the bivariate segmentation of SNP-array data for the characterisation of ASCNs in tumour samples. The formalisation generalises the problem and describes the extension from the univariate to the bivariate case, so further univariate methods can eventually be extended to the bivariate SNP-array case through such mathematical framework. With an appropriately selected Minkowski order, the generalisation considers the interaction between variables and their common features, but it is still capable of retrieving changes in a single variable. Thus, the proposed segmentation approach offers an intermediate stand between univariate approaches (e.g. CBS in GAP), which do not include the information available from both variables in the same model and are prone to skipping changes common to the two variables, and bivariate approaches with *p* = 1 (e.g. ASCAT), which overestimate the effect of variable interaction and tend to obviate single changes. The advantage of bivariate segmentation is more evident in low signal-to-noise ratio (SNR) scenarios, such as high degree of normal cell contamination and samples with high noise levels, where joint variable information reduces the chance of false positives and the promotion of single-variable changes avoids the reduction of recall rates. Additionally, in comparison to the univariate approach, duplicated estimation of change-points is avoided.

CnaStruct exemplifies the benefits of bivariate segmentation with adequately selected Minkowski order and outperforms existing methods at change-point detection on synthetic data. Besides, when coupled with the pattern recognition processes of GAP or ASCAT, the new workflows improve the downstream ASCN analysis in comparison to their original counterparts and the rest of compared methods. Notably, given its performance under the low contrast situations produced by high normal cell contamination levels and intra-tumour heterogeneity, CnaStruct should greatly improve allele-specific copy number characterisation in samples extracted from tumour biopsies, which are typically highly contaminated with normal cells, and in samples from advanced tumours, which are expected to present greater intra-tumour cellular heterogeneity.

## Competing interests

The authors declare no competing interests.

## Authors’ contributions

DMA conceived the study, supervised by AMA. Both authors participated in the writing of the manuscript. DMA devised the statistical model and performed the analyses. Both authors read and approved the final manuscript.

## Supplementary Material

Additional file 1 **Recall rates by normal cell contamination and alteration pattern, and alteration length for different parameterisations.** Recall rates (y-axis) by normal cell contamination level, sample pattern and alteration length (x-axis) for two different parameterisations of ASCAT (violet: default; brown: segmentation penalisation scaled by a factor of 0.35). Recall rates converge as region length increases, suggesting that both parameterisations achieve similar recall rates at long lengths, but the one that focuses on sensitivity is able to recall more short regions. Click here for file

Additional file 2 Description of the procedures to couple CnaStruct with GAP, ASCAT and TAPS. Click here for file

Additional file 3 **Recall rates by normal cell contamination and alteration pattern, and alteration length for assessed methods.** Recall rates (y-axis) of each of the assessed methods, calculated by normal cell contamination and alteration length (x-axis) over each of the five sample patterns. Colour code: purple (ASCAT), orange (GAP), black (GPHMM), blue (OncoSNP). Thicker lines correspond to the workflows in which CnaStruct was integrated. Click here for file

Additional file 4 **Results of the analyses of real data with a combination of CnaStruct and other methods.** The analyzed samples are: (i) Two samples from the Affymetrix platform, which are bundled with the TAPS software package (example02 and example16). These samples were analyzed with CnaStruct-TAPS. Provided TAPS results format: columns “Start” and “End” specify probe genomic positions within chromosome (ii) Two samples from the Illumina plaform, which come from a cell-line dilution series [8]. The two picked samples present normal cell contaminations of 0% and 53%. Chromosomes 6 and 16 were excluded beforehand (see [8,17]). These samples were analyzed with CnaStruct-GAP and CnaStruct-ASCAT. The compressed file contains one tab-delimited table per analysis. ASCAT results format: columns “start” and “end” specify probe indexes; “nA” and “nB” specify the number of A and B alleles, so the called copy numbers can be calculated from their sum. GAP results format: columns “Ind” and “Ind_K” specify probe indexes; “CN1” specifies the copy number. “Chromosome”; “Cn” specifies the copy number. Click here for file

Additional file 5 **Plots for the analysis of real data with a combination of CnaStruct and other methods.** The LRR profiles of several samples as analyzed with different combinations of CnaStruct and other methods are displayed. Colour code: blue, segment is called as being CN4 or higher; green, CN3; grey, CN2; red, CN1 or CN0. Only segments with more than 10 SNPs are superimposed. Even though ASCAT fails at the calling step on the 53% contamination sample, both ASCAT and GAP detect a loss on chromosome 13 not present in the pure tumour sample. Click here for file
