# Supplementary figures and images for: Bivariate segmentation of SNP-array data for allele-specific copy number analysis in tumour samples
Source: BMC Bioinformatics. 2013 Mar 5;14:84. doi: 10.1186/1471-2105-14-84 (PMC3599505; doi:10.1186/1471-2105-14-84)

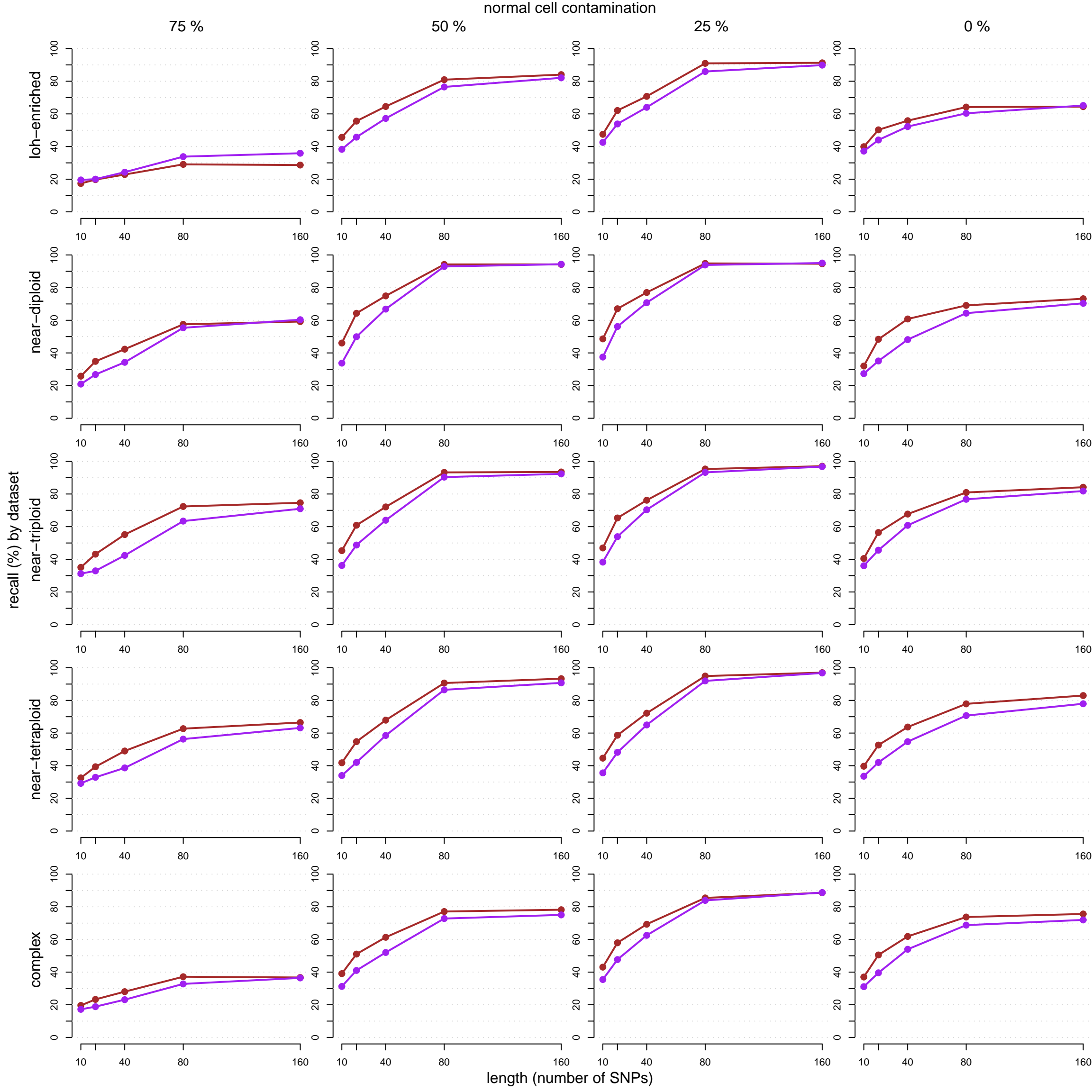

Supplement: Additional file 1 — Recall rates by normal cell contamination and alteration pattern, and alteration length for different parameterisations. Recall rates (y-axis) by normal cell contamination level, sample pattern and alteration length (x-axis) for two different parameterisations of ASCAT (violet: default; brown: segmentation penalisation scaled by a factor of 0.35). Recall rates converge as region length increases, suggesting that both parameterisations achieve similar recall rates at long lengths, but the one that focuses on sensitivity is able to recall more short regions. [file 1471-2105-14-84-S1.pdf]

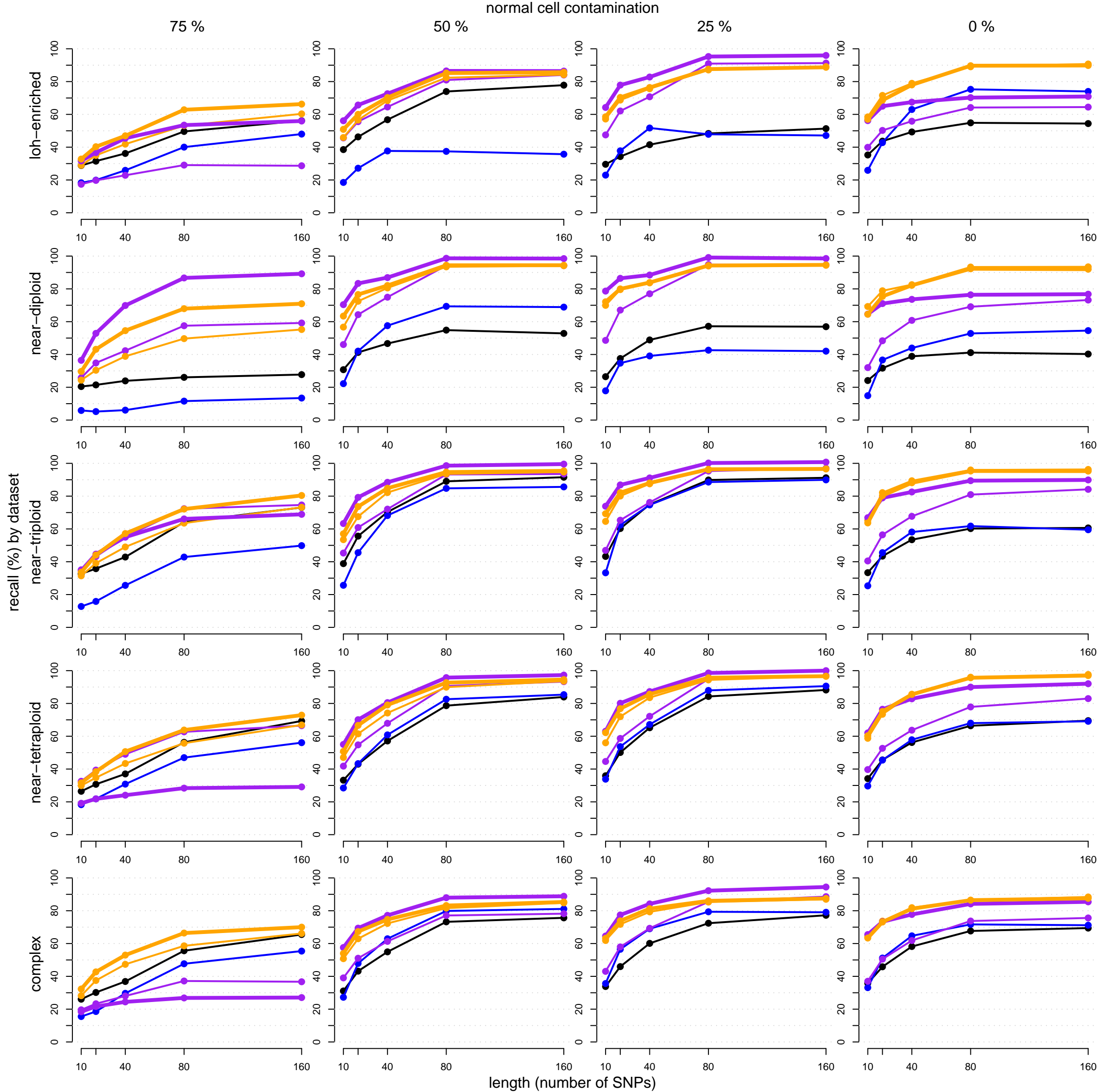

Supplement: Additional file 3 — Recall rates by normal cell contamination and alteration pattern, and alteration length for assessed methods. Recall rates (y-axis) of each of the assessed methods, calculated by normal cell contamination and alteration length (x-axis) over each of the five sample patterns. Colour code: purple (ASCAT), orange (GAP), black (GPHMM), blue (OncoSNP). Thicker lines correspond to the workflows in which CnaStruct was integrated. [file 1471-2105-14-84-S3.pdf]

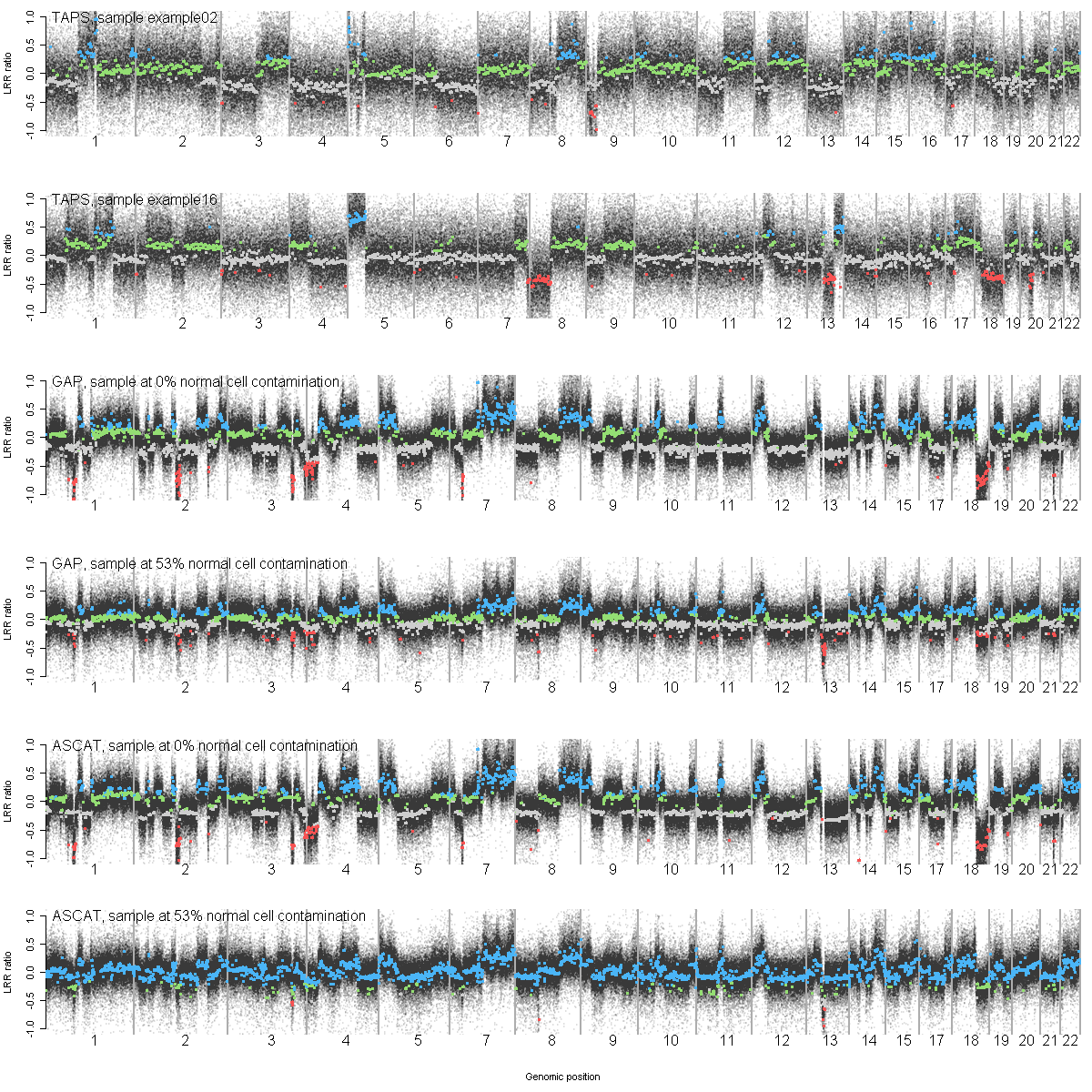

Supplement: Additional file 5 — Plots for the analysis of real data with a combination of CnaStruct and other methods. The LRR profiles of several samples as analyzed with different combinations of CnaStruct and other methods are displayed. Colour code: blue, segment is called as being CN4 or higher; green, CN3; grey, CN2; red, CN1 or CN0. Only segments with more than 10 SNPs are superimposed. Even though ASCAT fails at the calling step on the 53% contamination sample, both ASCAT and GAP detect a loss on chromosome 13 not present in the pure tumour sample. [file 1471-2105-14-84-S5.png]
