# Supplementary material for: Bivariate segmentation of SNP-array data for allele-specific copy number analysis in tumour samples
Source: BMC Bioinformatics. 2013 Mar 5;14:84. doi: 10.1186/1471-2105-14-84 (PMC3599505; doi:10.1186/1471-2105-14-84)
Supplement: Additional file 2 — Description of the procedures to couple CnaStruct with GAP, ASCAT and TAPS. [file 1471-2105-14-84-S2.doc]

# Coupling CnaStruct with ASCN analysis methods

## General changes

At the beginning of the file that you are required to change, add the following lines, adjusting the *meanSegLength* variable (minimum mean segment length after segmentation) as necessary.

library(CnaStruct)

meanSegLength = 100

## TAPS

Replace the *segment_DNAcopy* function with the new one.

segment_DNAcopy <- function(Log2) {

library(CnaStruct)

meanSegLength = 100

alf <- readAlf()

segs=NULL

chr_order = c(paste("chr",1:22,sep=""),"chrX","chrY","chrM")

chroms = chr_order[sort(which(chr_order%in%names(table(alf$Chromosome))))]

for (chrom in chroms) {

tlog = Log2[Log2$Chromosome==chrom,]

talf = tlog

talf$Value = NA

talf[which(talf$Start%in%alf[alf$Chromosome==chrom,2]),4] =

alf[alf$Chromosome==chrom,4]

if (nrow(tlog)>0) {

bps = breakpoints(tlog$Value, talf$Value,

maxseg=length(talf$Value)/meanSegLength, maxk=5*meanSegLength)

bpsStart = unique(c(1,bps))

bpsEnd = unique(c(bps,length(tlog$Start)))

output = as.data.frame(matrix(c(rep(chrom,length(bpsStart)),

tlog$Start[bpsStart],tlog$Start[bpsEnd],bpsEnd-bpsStart,

as.vector(unlist(lapply(1:length(bpsStart), function(i)

mean(tlog$Value[bpsStart[i]:bpsEnd[i]],na.rm=TRUE) )))),

ncol=5,byrow=FALSE))

segs=rbind(segs,output)

}

}

colnames(segs)=c('Chromosome','Start','End','Markers','Value')

return(segs)

}

## ASCAT

Replace the *ascat.aspcf* function in *ascat.R* with the new one.

ascat.aspcf = function(ASCATobj, selectsamples=1:length(ASCATobj$samples),

ascat.gg = NULL) {

attach(ASCATobj)

Tumor_LogR_segmented =

matrix(nrow=dim(Tumor_LogR)[1], ncol=dim(Tumor_LogR)[2])

rownames(Tumor_LogR_segmented) = rownames(Tumor_LogR)

colnames(Tumor_LogR_segmented) = colnames(Tumor_LogR)

Tumor_BAF_segmented = list();

for (sample in selectsamples) {

logRPCFed = bafPCFed = numeric(0)

tbsam = Tumor_BAF[,sample]

names(tbsam) = rownames(Tumor_BAF)

for (chrke in 1:length(chr)) {

lr = Tumor_LogR[chr[[chrke]],sample]

baf = tbsam[chr[[chrke]]]

bps = breakpoints(lr, baf,

maxseg=length(baf)/meanSegLength, maxk=5*meanSegLength)

logRPCFed = c(logRPCFed, smo(lr, bps))

mbaf = abs(baf-0.5)+0.5

mbaf[which(mbaf>=0.95)] = NA

bafPCFed = c(bafPCFed, smo(mbaf, bps))

}

names(logRPCFed) = rownames(Tumor_LogR)

names(bafPCFed) = rownames(Tumor_BAF)

bafPCFed = as.matrix(bafPCFed)

Tumor_LogR_segmented[,sample] = logRPCFed

Tumor_BAF_segmented[[sample]] = 1-bafPCFed

}

ASCATobj = list(Tumor_LogR = Tumor_LogR, Tumor_BAF = Tumor_BAF,

Tumor_LogR_segmented = Tumor_LogR_segmented,

Tumor_BAF_segmented = Tumor_BAF_segmented,

Germline_LogR=Germline_LogR, Germline_BAF=Germline_BAF,

SNPpos = SNPpos, ch = ch, chr = chr, chrs = chrs,

samples = colnames(Tumor_LogR))

detach(ASCATobj)

return(ASCATobj)

}

## GAP (original version)

Replace the *Step 3. Applying CBS algorithm to LRR and BAF profiles* section in the second script with the new code.

dataF[,C_smBAF]<-0

dataF[,C_bpBAF]<-0

mBAF<-dataF[,C_mBAF]*(1-sign(dataF[,C_gHomo]+dataF[,C_gLOH]))

bps = c()

st = 0

for (chr in names(table(dataF[,2]))) {

# print(chr)

sel = which(dataF[,2]==chr)

bps_chr = breakpoints(dataF[sel,C_LRR], mBAF[sel],

homthr=germHomozyg.mBAF.thr,

maxseg=length(sel)/meanSegLength, maxk=5*meanSegLength)

bps = c(bps, bps_chr + st)

st = st + length(sel)

# print(length(bps_chr))

}

dataF[bps,C_bpBAF]<-1

dataF[bps,C_bpLRR]<-1

bps = unique(c(1,bps,length(dataF[,C_LRR])))

for(k in 1:(length(bps)-1)){

dataF[bps[k]:bps[k+1],C_smLRR]<-

median(dataF[bps[k]:bps[k+1],C_LRR],na.rm=T)

dataF[bps[k]:bps[k+1],C_smBAF]<-

median(dataF[bps[k]:bps[k+1],C_BAF],na.rm=T)

}
